# Glottal stops do not constrain lexical access as do oral stops

**DOI:** 10.1371/journal.pone.0259573

**Published:** 2021-11-19

**Authors:** Holger Mitterer, Sahyang Kim, Taehong Cho

**Affiliations:** 1 University of Malta, Msida, Malta; 2 Hanyang Institute for Phonetics and Cognitive Sciences of Language, Hanyang University, Seoul, Korea; 3 Hongik University, Seoul, Korea; 4 Department of English Language and Literature, Hanyang University, Seoul, Korea; Universita degli Studi di Milano-Bicocca, ITALY

## Abstract

This study explores processing characteristics of a glottal stop in Maltese which occurs both as a phoneme and as an epenthetic stop for vowel-initial words. Experiment 1 shows that its hyperarticulation is not necessarily mapped onto an underlying form, although listeners may interpret it as underlying at a later processing stage. Experiment 2 shows that listeners’ experience with a particular speaker’s use of a glottal stop exclusively as a phoneme does not modulate competition patterns accordingly. Not only are vowel-initial words activated by [ʔ]-initial forms, but /ʔ/-initial words are also activated by vowel-initial forms, suggesting that lexical access is not constrained by an initial acoustic mismatch that involves a glottal stop. Experiment 3 reveals that the observed pattern is not generalizable to an oral stop /t/. We propose that glottal stops have a special status in lexical processing: it is prosodic in nature to be licensed by the prosodic structure.

## 1. Introduction

Spoken word recognition requires listeners to map acoustic phonetic input onto stored information about the words of their language. There is, however, considerable disagreement about memory storage for the words of a language—i.e., how words are stored in the mental lexicon and what kinds of information are stored about those words. Some theories assume that words are stored in the form of abstract representation (e.g.,[1]), whereas others assume that they are stored with details about multiple phonetic forms or exemplars of each word [2–4]. A hybrid position could be that listeners store multiple but abstract forms of each word (e.g., [5]). In the present study, we provide evidence that different phonemes can constrain lexical processing differently, which informs the theoretical debates about how words are stored with what kinds of word information in the mental lexicon.

Assumptions about the storage of words in the mental lexicon have immediate consequences for assumptions about processing of acoustic-phonetic input. This is because the input is generally assumed to be processed in terms of how it matches the stored information of some sort about a word in the lexicon, thus determining lexical access. That is, the output of pre-lexical processing (or the processing of acoustic-phonetic input at an early stage) and the lexical representation need to be commensurable. Some researchers argue that such a matching process must involve some form of abstraction [6], so that the mental lexicon contains sequences of abstract units, potentially including allophones [7, 8]. One classic assumption regarding this matching process is that an initial mismatch (i.e., when the onset of the acoustic input is mismatched with the stored speech information about the onset of a potential lexical candidate) leads to strong deactivation of lexical competitors [9, 10]. The initial (onset) mismatch constrains lexical activation so strongly that, for example, the original Shortlist model [11] assumes that its deactivation effect would require at least three subsequent matching (overlapping) phonemes to be counteracted. The importance of the onset overlap is clear in eye-tracking data which demonstrate that competitors with an initial overlap (such as *beaker* and *beetle*) receive much more looks than competitors with a final overlap (such as *beaker* and *speaker*), even though the latter pair overlaps more in the number of phonemes than the former [9, 12]. The multiple activation effect of the onset overlap can be reduced when uncertainty arises through either extraneous noise or a casual speech style [13, 14].

Recently, however [15], investigated lexical competition patterns in Maltese and reported some surprising results. The study focussed on glottal stop-initial words to examine the dual function of the glottal stop in Maltese, i.e., as part of its phonemic inventory and as an epenthetic glottal marker of a prosodic boundary. An eye-tracking experiment was performed to investigate the competition between glottal-stop-initial words (such as *qattus* /ʔɑtːʊs/, Engl. ‘cat’) and vowel-initial words (such as *attur* /ɑtːʊr/, Engl. ‘actor’) that overlapped considerably in their segmental make-ups (e.g., both contained the sequence /ɑtːʊ/). Given the pattern of segmental overlap in the two words (i.e., with the onset mismatch due to the presence or absence of a glottal stop), a general expectation would be that they would show a lexical competition effect similar to that of *beaker* and *speaker* in English. [15] also considered the fact that vowel-initial words in Maltese (as can be the case in other languages) are often marked by an epenthetic glottal stop that can serve as a phonetic marker of a prosodic boundary aligned with the onset of vowel-initial words. Such an epenthesis occurs in about 50% of the eligible cases [see 15], and when it occurs, the epenthetic glottal stop is phonetically very similar to an underlying glottal stop (i.e., /ɑtːʊr/ → [Ɂɑtːʊr]), so that, with a database of more than 800 tokens, underlying and epenthetic glottal stops were not distinguishable by acoustic measures such as duration and quality of the glottal stop (i.e., whether a full closure was achieved or not). This is noteworthy and distinguishes the case of the epenthetic glottal stop in Maltese from other types of epenthetic segments that might occur at word boundaries in other languages. For instance, in English, an epenthetic linking [r] and an underlying /r/ are differentiated in fine phonetic detail [16] to which listeners are sensitive. A similar case may be found with the fine phonetic difference between an underlying /r/ and a liaison /r/ in French [17].

The results in Maltese from [15] showed that listeners considered both lexical hypotheses—the glottal stop-initial and vowel-initial words—to the same extent until later segments distinguished them. For example, upon hearing [Ɂɑtː], listeners looked at both *qattus* and *attur* to a similar extent, and eventually looked at the target word only as cued by the final segment [r] or [s]. It was a surprising result, given that the initial [Ɂ] in the phonetic input is acoustically consistent with only one of the competing lexical hypotheses at the abstract lexical level: *qattus* /ʔɑtːʊs/.

One way to explain this finding would be that apparently vowel-initial words such as *attur* contain an underlying glottal stop, and there is only a difference in the orthography. However, there are phonological processes that indicate that this is not the case. When consonant initial words are preceded by the Maltese definite article *(i)l*, the [ɪ] in the article surfaces (e.g., *il-palk* [ɪlpɐlk], Engl., ‘the stage’). This also happens for glottal stop-initial words (*il-qattus* [ɪlɁat:ʊs], Engl., ‘the cat’), but for vowel-initial words, the article attaches to the word and the initial /ɪ/ is dropped (*l-attur* [lat:ʊr], Engl., ‘the actor’). This also happens when another preposition is added (*bil-qattus* [bɪlɁat:ʊs], Engl. ‘with the cat’ vs. *bl- attur*[blat:ʊr], Eng., with the actor). This indicates that not only is there a difference in the orthography but also the underlying phonological representations differ between word-initial words and glottal stop-initial word.

Given that vowel-initial and glottal stop-initial words are hence distinct in their phonological representation, the findings of [15] in their eye-tracking task stands in sharp contrast to the general assumption that listeners prefer to interpret an input signal as being phonetically faithful to an underlying phonological representation (or phoneme). For instance, when hearing a nasalized vowel, native listeners of Hindi, a language in which vowels are phonologically contrastive in nasality but also allow for coarticulatory nasalization, tend to interpret nasality as stemming from an underlying nasal vowel rather than from a contextually-driven coarticulatory nasalization of an oral vowel [18]. A similar effect was found with Dutch listeners interpreting a surface form of an approximant [19]. A bilabial approximant that appears on the surface level in Dutch can come from either an underlying approximant or a bilabial stop /b/ that can be phonetically lenited to become an approximant. When presented with versions of /b/-initial words with /b/ produced as a labial approximant, Dutch listeners consistently preferred target words with an underlying approximant, despite the fact that the surface form of the approximant was derived from an underlying stop.

The processing pattern observed with the glottal stop in Maltese clearly deviates from these patterns. Maltese listeners do not attribute the phonetic form of a word-initial glottal stop faithfully to an underlying (phonemic) glottal stop. Mitterer et al. [15] explored whether the lack of the phonetic correspondence effect in the processing of a glottal stop in Maltese can be understood in connection with the processing of prosody (or a prosodic boundary), which is assumed to influence lexical competition (e.g., [20]). This question was motivated by the production pattern that an epenthetic glottal stop with a vowel-initial word is more likely to occur at a larger prosodic boundary, as is found in English [21]. Mitterer et al. [15] indeed showed that prosodic processing (i.e., computing a prosodic boundary in this case) influences the decision on whether a glottal stop is phonemic or epenthetic: Listeners were more likely to interpret a glottal stop as epenthetic when the prosodic cues were consistent with a larger prosodic boundary. It is interesting to note that the prosodic effect was observed only at a later processing stage, indicating that the initial segmental analysis was further modulated by the prosodic analysis that comes into effect relatively later in lexical processing [see 22, 23, for related results and discussion]. Crucially, however, their time-course data for speech processing showed evidence that, upon hearing a glottal-stop-initial word, listeners activated a vowel-initial word relatively *early* in the processing stage, independent of prosodic boundary conditions. Based on these results, Mitterer et al. [15] concluded that vowel-initial words are stored with phonetic variants that contain a glottal stop, and hence a vowel-initial word remains activated even when the speech signal is acoustically consistent with an underlying glottal stop.

In this study, we build on [15] and continue to explore the nature of the rather surprising lexical competition effects observed with Maltese glottal stops. We conducted three experiments. In Experiment 1, we explore whether and how the fine phonetic detail of a glottal stop can influence the lexical processing of two competing word sets (/Ɂ/-initial words vs. V-initial words). Given that a hyperarticulated form of a segment is generally assumed to enhance the phonological (underlying) representation of the segment [e.g., 24–27], we test whether the heightened phonetic clarity that arises with a hyperarticulated form of a glottal stop facilitates the perception of the underlying phonemic representation more than that of a glottal stop variant that is assumed to be stored along with a vowel-initial word in the lexicon.

Experiment 2 is based on the finding that speakers vary in the use of an epenthetic glottal stop for a vowel-initial word. We investigate whether the unusual processing pattern for a glottal stop (especially the strong activation of vowel-initial words with the speech stimuli that contain an initial glottal stop) observed in Experiment 1 of the present study and [15] could be due to the distribution of experimental stimuli that consistently contained an epenthetic glottal stop in the vowel-initial target word. Therefore, we add an exposure phase during which participants heard the speaker producing vowel-initial words without glottalization, and all vowel-initial words in the main experiment are produced without a glottal marking. We test whether listeners can adapt to this and activate vowel-initial words less when hearing a glottal gesture. Testing this possibility also addresses a more general question of how listeners adapt to a given speaker [28–30], which has been observed for connected-speech patterns [19] and for prosodic patterns that vary across speakers [31]. Finally, in Experiment 3, we follow up the results of Experiments 1 and 2 to compare how an oral stop constrains lexical access (/t/-initial words versus V-initial words with a similar number of overlapping phonemes) with how a glottal stop does it in Maltese. We conduct this experiment to confirm the special status of the glottal stop whose constraint on lexical access is assumed to differ from that of other segments.

## 2. Experiment 1

In this experiment, we test whether the strength of a glottal stop influences listeners’ interpretation of the stop as epenthetic or underlying. This question was motivated by [15]’s findings. They examined the production data of 16 participants (with 35 observations per condition) and revealed that the frequency of occurrence of the glottal stops at the surface was far greater in the underlying condition (ca. 97%) than in the epenthetic condition (ca. 50%) with the location of stress matched within pairs (see the R-markdown file for the production study by [15] at OSF https://osf.io/wdjhz/.) Note that by comparison [32], used 16 participants and 24 observations per condition to assess incomplete neutralization in German word-final stops and found consistent effects that they described as quite subtle. Crucially, however, the surface forms of the glottal stops showed no significant acoustic differences between the two conditions in terms of the distribution of the phonetic variant type of glottal gesture (a full glottal stop versus glottalization) and the duration of the glottal gesture. Given the acoustic similarities of glottal stops from difference sources in terms of duration and quality (as measured by [15]), it is not clear how listeners discern the two sources. It is, however, worth noting that glottal stops from different sources might be further differentiated by other phonetic correlates such as spectral tilt and noise measures [33]. So it remains to be seen whether the seemingly similar phonetic forms from difference sources can be differentiated by other phonetic cues.

As a first step to explore this question, we test whether a hyperarticulated version of a glottal stop with a full closure leading to a period of silence in the signal may lead to different perceptual interpretations. Existing research could motivate different hypotheses. On the one hand, listeners might prefer to interpret the hyperarticulated phonetic form of a glottal stop as associated with an underlying glottal stop rather than an epenthetic one. This is because the hyperarticulated form is generally assumed to enhance the distinctive phonological features of a segment, which in turn leads to maximization of lexical distinction [see ch. 7 of 34 for related discussion].

Given that the [glottal] feature is taken to be the distinctive feature of the underlying glottal stop, its hyperarticulation will provide stronger phonetic support for an underlying glottal stop than for an epenthetic one. Moreover, as discussed in [15], the equally strong lexical competition between /Ɂ/-initial words and V-initial words implies that listeners are uncertain about the physical presence of a glottal stop in the input signal. Taken together, the evidence leads to the prediction that listeners will prefer to interpret a hyperarticulated glottal stop (i.e., with a full glottal closure) as an underlying glottal stop.

However, these considerations are contingent on whether a glottal stop with a full closure is indeed a hyperarticulated phonetic form that enhances its underlying representation as just discussed above [24–27]. In fact, as was noted by Davidson [35], it is not often the case that a glottal stop is realized with a full closure across languages [see also 36, 37]. For example [35], showed that a phonemic glottal stop in Hawaiian is most often realized as creaky voice especially in a word-medial intervocalic context with flanking vowels being ‘glottalized.’ Interestingly, a glottal stop with full closure in Hawaiian was found to occur more likely in word-initial position, which [35], suggested, may be related to recoverability and segmentation. [35] therefore leaves a question open as to “what the gestural target for this phoneme is”—i.e., a glottal closure versus a period of creaky voice. Garellek et al. [36] suggested yet another option that the target is simply specified with [glottal constriction] which may or may not lead to full closure depending on the prosodic strength of the gesture. This debate, however, does not entirely rule out a possibility that the non-modal creaky voice in an intervocalic context can reflect a form of lenition of a full glottal gesture while a full glottal stop reflects an underlying gestural target.

Davidson’s observations [35] also relate to a wider debate about a tug of war between frequency of occurrence and the prototypicality of speech signals. In fact [37], also noted that the full stop is not the most frequent implementation of the glottal stop across languages. This is somewhat similar to, for instance, word-medial /t/ in English, which is also unlikely to surface as a full stop. However, while frequency of a form generally predicts ease of recognition [e.g., 38], the full stop for word-medial /t/, though *infrequent* in word-medial position, leads to a generally better recognition [38, 39]. Moreover, our previous production study [15] indicates that the full stop in Maltese is not that infrequent, accounting for around 60% of the cases in which there is a glottal gestures, independent of whether that is triggered by an underlying or an epenthetic glottal stop in phrase-medial position. Finally [40], investigated the realization of word-medial glottal stop in singleton and geminates and found that, while the glottalization is the dominant form for geminates, the full stop is the dominant form for geminates. Since geminates are often considered a strong from of a given segment, it is therefore reasonable to assume that a full stop at least in Maltese is a strong phonetic form of a glottal gesture associated with the underlying phoneme.

Alternatively, listeners might associate the assumed hyperarticulated form of a glottal stop equally with a variant form of a vowel-initial word. This possibility is related to [15]’s view that phonological variants of vowel-initial words produced with a glottal stop are stored in the mental lexicon. Under this view, we cannot rule out the possibility that the hyperarticulated form of a glottal stop will also provide phonetic support for the lexically represented (stored) variant of a glottal stop-initial form of a vowel-initial word. Such phonetic support for a vowel-initial word can be as strong as that for an underlying glottal stop-initial word, in which case the hyperarticulated form will not lead to any preference for one over the other. We use an eye-tracking experiment to test these possibilities.

### 2.1 Method

#### 2.1.1. Participants

Thirty-two students at the University of Malta participated in this experiment. They were native speakers of Maltese and participated for a small monetary compensation. The participants were 19 women and 13 men, aged 18 to 33 years.

#### 2.1.2. Apparatus

Experiments were performed in a sound-attenuated booth at the Cognitive-Science Lab at the University of Malta. They were run on a standard PC using Experiment Builder, and eye movements were tracked with an Eyelink 1000 eye-tracker in desktop mode at a frequency of 500Hz.

#### 2.1.3. Stimuli

The stimuli were based on those used by [15] in their third experiment (materials available at: https://osf.io/wdjhz/), which also used a visual-world paradigm with written words as targets to click on. An adult female speaker of Maltese produced multiple renditions of sentences that took the form *[Matthew|Daniel|Mary|Jenny] [j|t]ifhem TARGET* (Engl. ‘[Matthew|Daniel|Mary|Jenny] understands TARGET’). The alteration between *jifhem* and *tifhem* was necessary because Maltese uses different forms for the masculine and feminine third person (i.e., *Matthew jifhem* vs. *Jenny tifhem*). Thus, each target word occurred in phrase-medial position preceded by a /m/-final word.

**Fig 1:**
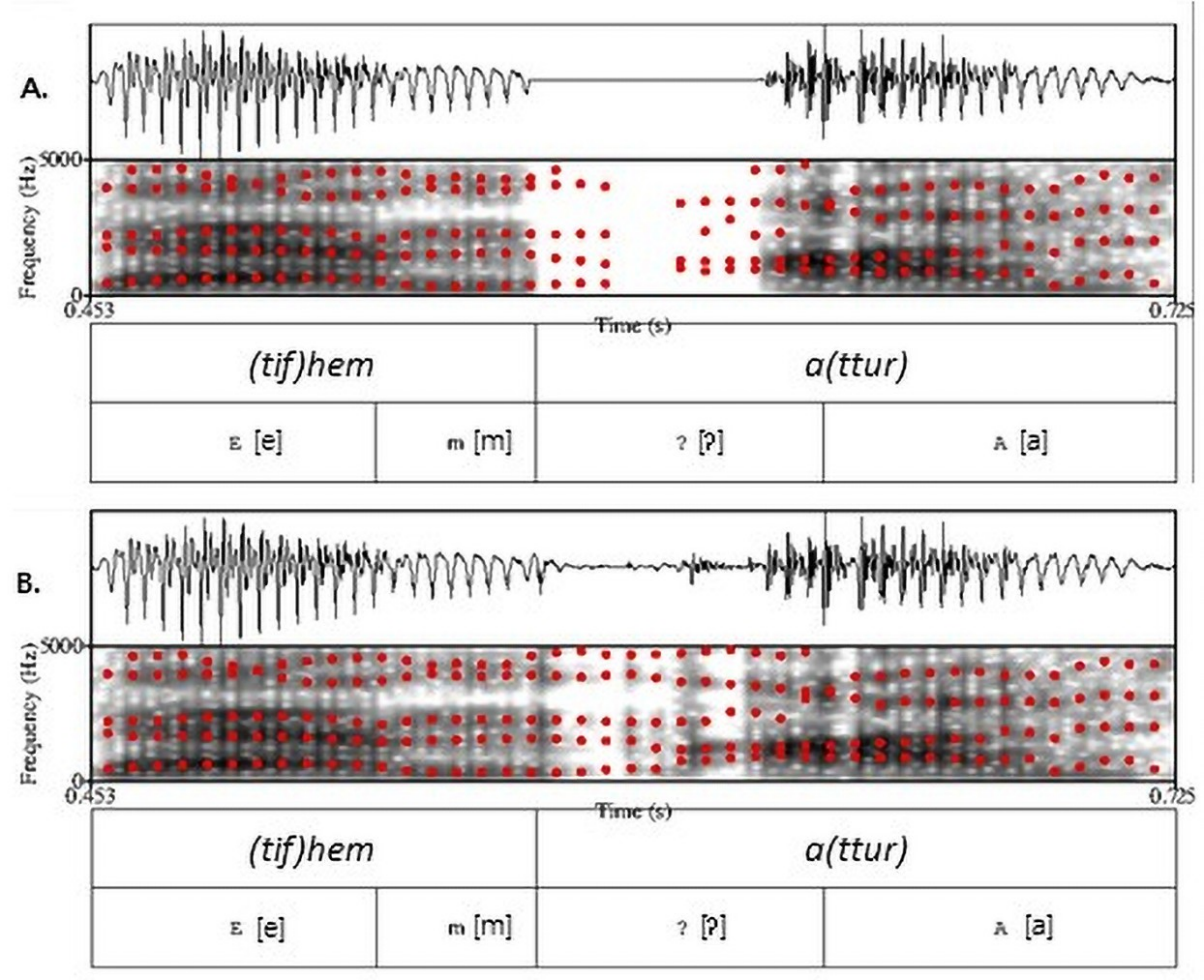
Oscillogram and spectrogram with estimated formant locations (5 formants assumed till 5kHz) at the critical juncture of one experimental item (*attur*, Engl., ‘actor’) with glottal stop (A) and glottalization (B).

We used 48 pseudo onset-overlap pairs of vowel-initial words and glottal stop-initial words from [15] in the version with just a prosodic word boundary (i.e., no phrase-level boundary) between the target word and the preceding *[j|t]ifhem*. To generate versions with a full glottal stop, we replaced the initial glottalization (with a mean duration of 58 ms and a range from 31 to 80 ms) with a full silence for both the underlying and epenthetic glottal stops. To prevent clicks, there was a 5 ms ramp down into the silence and 5 ms ramp up to the original signal after the silence. In doing so, care was given not to cut off the portion of the formant transitions, so that the full silence was still consistent with realization of a full glottal stop which is often accompanied by some degree of glottalization during the vowel [36]. The same 120 filler trials were used as in [15], with the target words of those trials using neither a vowel- nor glottal stop-initial. Fig 1 shows an example with the target word *attur* (Engl., ‘actor’) with glottalization and a full glottal stop.

For the visual display, unrelated distractor words were added to the critical pseudo onset-overlap pairs so that there were four words on the screen. Filler trials were generated to discourage participants from trying to guess the target. For instance, forty targets were accompanied by onset-overlap pairs (e.g., *ballun-baliena*, Engl., ‘ball’-‘whale’) plus an unrelated distractor on the screen to prevent participants from assuming that, if there were two phonologically similar words on the screen, one of them was likely to be the target. For the remaining 80 filler trials, a vowel-initial word or a glottal stop-initial word was used as one of the distractors, again, to discourage participants from assuming that any vowel-initial or glottal stop-initial word on the screen was likely to be the target.

#### 2.1.4. Procedure

Participants first read an instruction that familiarized them with the visual-word paradigm. They were instructed to click on the word that “was understood,” that is, the object (TARGET) of the sentence *[Matthew|Daniel|Mary|Jenny] [j|t]ifhem TARGET*. After they read the instructions, the eye-tracker was calibrated using a nine-point calibration, and then the main experiment began.

Each participant completed 168 trials (48 experimental trials and 120 fillers). The experiment started with 3 filler trials. A different random order was generated for each participant, with the following constraints: each critical pair was presented once, and the condition for that pair was counterbalanced across participants. The targets were rotated over four conditions (crossing the two target types, V versus /ʔ/ with the phonetic implementation of full stop versus glottalization). Moreover, the target and competitor positions were counterbalanced for each participant, so that each of the 12 possible combinations for the target and competitor positions occurred once in each experimental condition and 10 times in the 120 filler trials. We did that to ensure that participants’ preference to start scanning at the upper left corner of the screen did not influence the results. After every 50 trials, participants were told how many trials they had completed and had the opportunity to take a short break.

On each trial, participants saw the display for 2s before the sentence started, and there the initial part up to the target word had a duration that ranged from 0.57 to 0.67s. A trial only ended when participants clicked on one of the words on the display (though clicks were accepted when made anywhere on the screen). The click ended the trial unless the soundfile was still playing. The inter-trial-interval between the end of one trial (either through the click or the end of the soundfile in case of an early response) and the start of the next trial was 0.6s.

This study was reviewed and approved by the committee of the internal review board of HICPS (Hanyang Institute for Phonetics & Cognitive Sciences of Language). The participants have signed a consent form to participate in the research; and the acoustic data were analysed anonymously.

### 2.2. Results

We analysed the click responses and eye-movements in trials with a correct response. For the eye-tracking data, missing data for blinks and saccades were replaced by those from the preceding location (for blinks) and following fixation (for saccades). Table 1 provides descriptive data for both accuracy and latency of the click responses. There were few errors, with accuracy in all conditions in the range of 98.1% to 98.9%. Given the narrow range and the ceiling effect, it is not useful to statistically test for differences between the conditions in terms of accuracy. For the latency of the click responses (calculated from the onset of the target word), we used linear mixed-effect models with the logarithm of the reaction time as the dependent variable. The first model estimated the reaction time based only on participants and items (i.e., rt ~ 1 + (1|participant) + (1|item)). Trials with a residual larger than 2.5 standard deviations from the mean in this model were rejected for further analysis (42 trials, 2.73% of the data). A second linear mixed-effect model then used contrast-coded predictors of segment (V = -0.5, /ʔ/ = 0.5) and realization (glottalization = -0.5, full stop = 0.5) and their interaction as fixed factors.

**Table 1:** Mean accuracy in % and reaction time in ms (SD for single trials in brackets) for Experiment 1.

	Full Stop	Glottalization	Total
/ʔ/-initial	98.70 / 1418 (452)	98.18 / 1365 (441)	98.44 / 1391 (447)
V-initial	98.44 / 1453 (461)	98.96 / 1434 (462)	98.70 / 1443 (461)
Total	98.57 / 1435 (457)	98.57 / 1400 (453)	98.57 / 1418 (455)

The model was initially specified with a full random effect structure, but it only converged when all random slopes had been removed. The final model revealed that reactions were significantly faster with a glottalization than with a full stop in the signal (b = 0.027, SE = 0.013, t(1367) = 2.075, p = 0.038). The effect of target type was only marginally significant (b = -0.038, SE = 0.02, t(92) = -1.833, p = 0.070), and the interaction was not significant (b = 0.018, SE = 0.026, t(1367) = 0.728, p = 0.466).

**Fig 2:**
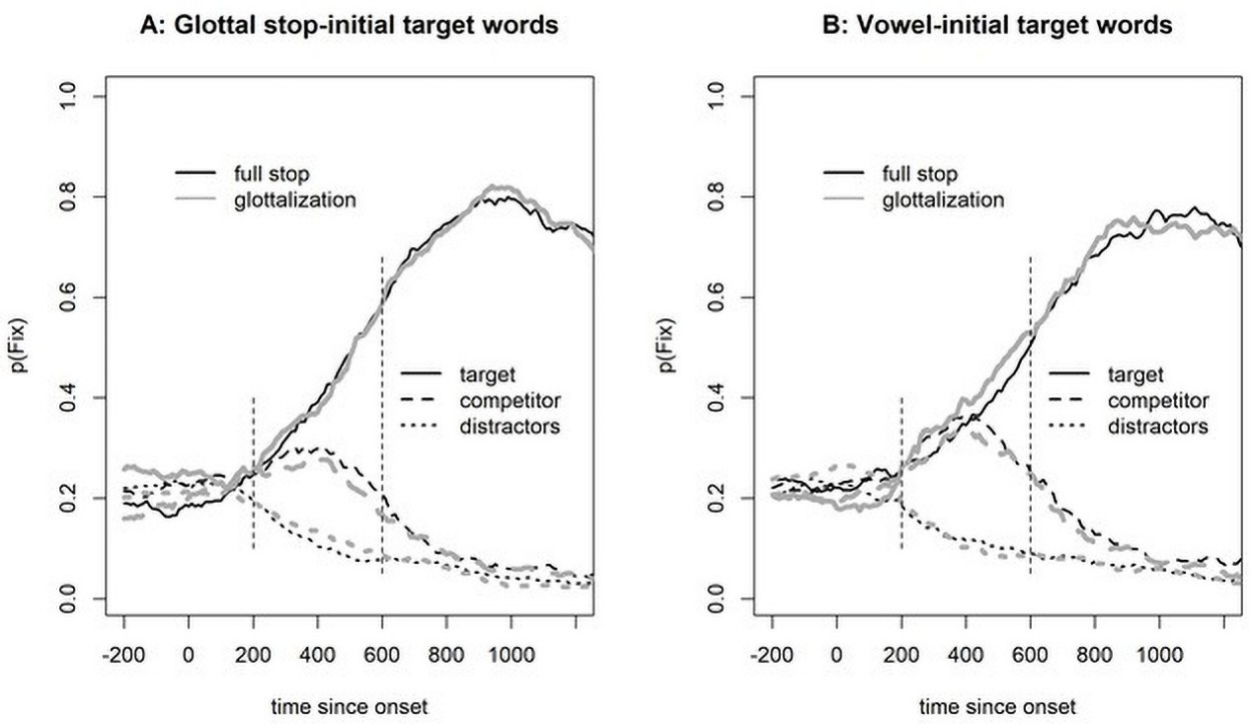
Fixation proportions in Experiment 1. Note that the vertical dotted lines indicate the analysis window, 200–600 ms after target-word onset.

Fig 2 shows the fixation proportion on correct trials in Experiment 1. For both glottal stop-initial and vowel-initial targets, we see an initial parallel rise for target and competitor. However, for glottal stop-initial targets, competitor fixations decrease sooner (around 300 ms) than for vowel-initial targets (around 400 ms).

For statistical analysis, we calculated a measure of target preference by subtracting the logOdds of competitor fixations from the logOdds of target fixations at 200–600 ms after onset of the target word, a time window that indicates initial lexical access [following 15]. The contrast coding was the same as for the reaction time analysis (target onset: (V = -0.5, /ʔ/ = 0.5; realization: glottalization = -0.5, full stop = 0.5)). The analysis started with a full model, but only the random intercept-only model converged. This model revealed a marginally significant effect of target onset (b = 0.522, SE = 0.275, t(94) = 1.901, p = 0.060), no effect of realization (b = -0.285, SE = 0.215, t(1379) = -1.324, p = 0.186), and no significant interaction (b = 0.234, SE = 0.430, t(1380) = 0.544, p = 0.586).

**Fig 3:**
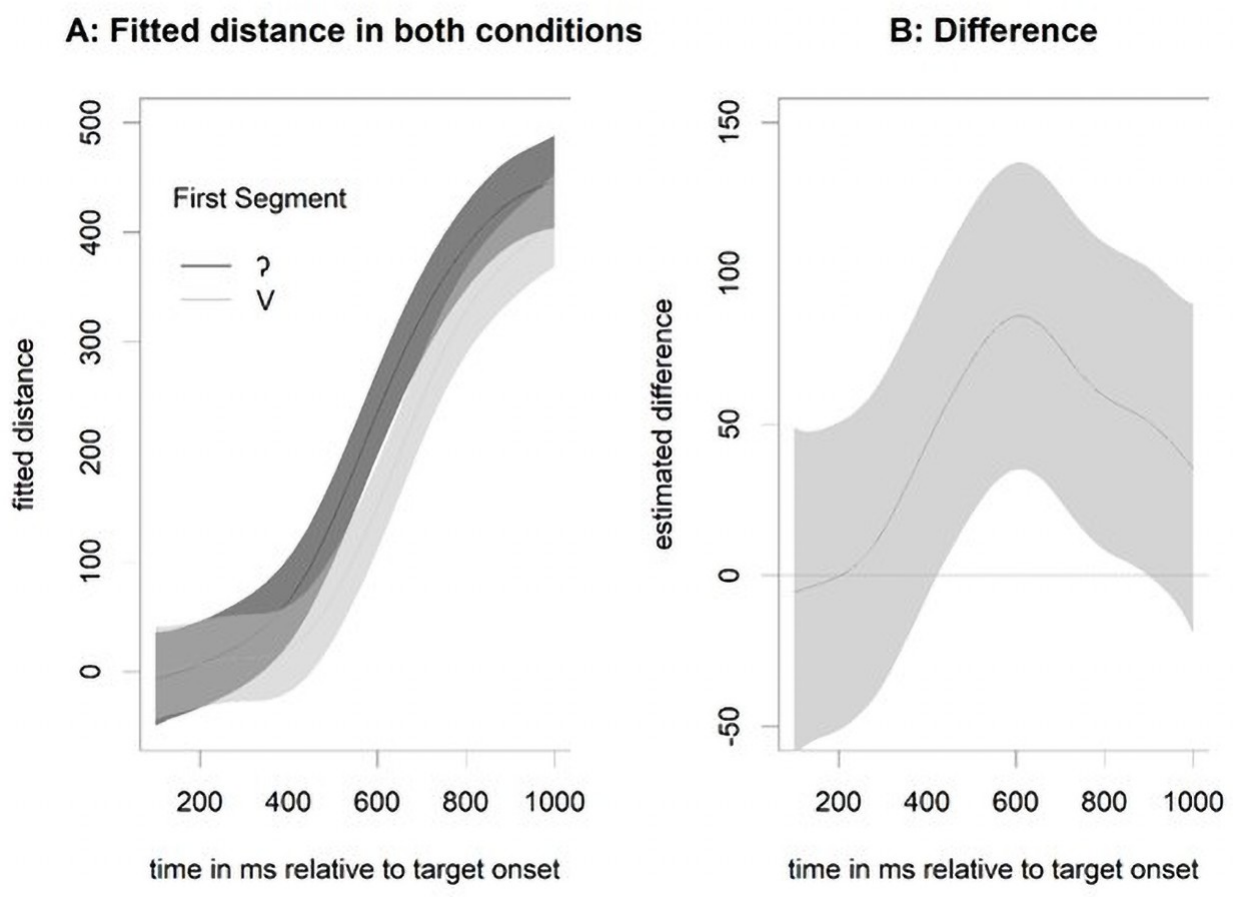
Outcome of the general additive model for target preference comparing vowel-initial words with glottal stop-initial words.

Given the marginally significant effect of target in both the reaction time and the fixation analyses, we performed an additional exploratory analysis using a general additive model (GAM) to investigate where in the time series the two conditions differed significantly (along the lines of [35]). Since a GAM analysis requires a continuous variable, we calculated the target preference with the Euclidean distance (cf., [36]) from the fixation position to the competitor and to the target (preference = distance(fixation,competitor)–distance(fixation,target)). We capped these values at 600 pixels because differences larger than that were due to the layout of the display on any given trial (i.e., the distance is larger if the target and competitor are at opposite sides of the screen but smaller if one is above the other). The first model with a full random effect structure (i.e., a smooth term for both participants and items, with a random slope for target onset over participants) was calculated to get an estimate of the autocorrelation in the data (found to be 0.955). A second model was run with an autocorrelation correction (which was successful because there were no autocorrelations at lags larger than zero). Fig 3 shows the outcome of the GAM, with a significant difference between the conditions starting at 427 ms and ending at 890 ms, that is, when the confidence interval does not include zero.

### 2.3. Discussion

The results of Experiment 1 indicate that the two competing words (/Ɂ/-initial versus V-initial) are activated to an equal degree, regardless of the fine phonetic detail of the glottal stop—i.e., whether it is a hyperarticulated phonetic form (with complete glottal closure) or not. The results therefore do not support the hypothesis that Maltese native listeners prefer to associate a hyperarticulated glottal stop with an underlying glottal stop rather than with an epenthetic one. It appears that listeners do not make reference exclusively to the underlying phonological features when processing the surface form of a glottal stop, but instead, simply follow the statistical regularities of the input, which also provide equal support for a variant form (initial glottal stop) of a vowel-initial word. This finding runs counter to the general assumption that a hyperarticulated phonetic form enhances the distinctive feature of a segment which in turn augments lexical distinction [24–27].

These results again allude to another possible conceptualization of the contrast between vowel-initial and glottal stop-initial words. On the one hand, given the frequent occurrence of glottalization or a glottal stop at the start of orthographically vowel-initial words, vowel-initial words may be considered as glottal stop-initial specified in its underlying phonological representation. On the other hand, the letter <q> in a supposedly glottal stop-initial word may indicate that it was historically derived from Arabic uvular stop ‘q’, which may have undergone a sound change from *q > ʡ [cf. 41]. One might therefore hypothesize that the assumed underlying glottal stop-initial word is in fact an underlying epiglottal stop. If it is the case, the contrast between the orthographically vowel-initial and <q>-initial words in Maltese would be the contrast between a glottal stop versus an epiglottal stop in the phonological representation. Such a phonological difference should then be differentiated by phonetic implementation. For instance, Esling et al. [42] suggest that epiglottal stops have more overall constriction than glottal stops, which could translate to a distinction in Maltese between (mostly voiced) glottalization for orthographically vowel-initial words and a full (epi)glottal stop for orthographically <q>-initial words.

This possibility, however, runs counter to the two related phonetic and phonological facts. First, the results of our previous phonetic production study [15] show that when there is an epenthetic glottal stop for vowel-initial words, it is realized as a full glottal stop as frequently as with the case with an underlying glottal stop. When considering the difference reported in [43], the epenthetic glottal stop, with glottalization as the phonetic target, should be produced with such glottalization more often than words with an underlying full glottal stop. Second, as we explained in the introduction, there is a phonological process that clearly suggests that orthographically vowel-initial words are indeed vowel initial. Recall that the [l] in a preceding definite article (/il-/) is syllabified as an onset with the vowel-initial word without a realization of the preceding vowel /i/ in /il-/ as in *l-attur*, whereas a consonant-initial word does not take [l] as its onset, so that [l] is syllabified with a preceding [i] as in *il-qattus*. This suggests that the orthographically vowel-initial word does not contain any consonant in its underlying phonological representation. In addition to these facts, if the orthographically <q> -initial words were indeed associated with a full glottal stop, being phonetically differently from a glottalization for orthographically vowel-initial words, one should be able to observe differential competitions as a function of the phonetic detail for a vowel-initial word versus a glottal stop-initial word. Our results here, however, indicate that the pattern of competition is not modified by phonetic detail at all, regardless of whether the glottal feature is realized as a full stop or glottalization. This also takes support away from the hypothesis that orthographically vowel-initial words in Maltese are phonologically specified with glottalization and <q>-initial words with a full glottal stop. It is also worth mentioning that while the form of the glottal gesture (full stop vs. glottalization) did not modify the pattern of competition between glottal stop-initial and vowel-initial words, recall that there was an effect of this variable on reaction latency. This might be explained by the fact that glottalization is more informative than a full closure, presumably because glottalization can only arise from a glottal gesture, whereas a full stop is also congruent with other oral stops produced with a full closure. This might explain the finding that glottal-stop signals (with a full closure) lead to slower reaction times.

The results also do provide some new data that merit further discussion. In contrast to the results of [15], these data show some evidence that listeners prefer to interpret /Ɂ/-initial words in a phonetically faithful way as corresponding to an underlying glottal stop. This preference is not evident in an early analysis time window, but it arises in a later analysis time window at around 400ms after the onset of the glottal stop. If we consider the processing latency which means that the influence of speech input is likely to be observable with eye-movements at 150–200 ms after the onset of the pivotal speech sound is heard, the effect we observed at around 400 ms indicates that the perceptual preference comes into effect about 200 ms after the initiation of lexical access. This time course, with a later effect of segmental information, contrasts with the general perceptual impact of other types of speech input that occurs early in lexical processing. Lexical effects [44], auditory contrast effects [45], and phonological learning effects [46] have all been found to occur in early stages of speech processing.

An explanation can be offered here as to why the current case deviates from the general pattern. A glottal stop at the onset of a word might be processed differently from other segments for two reasons. First, a glottal stop with no information about the constriction location in the vocal tract (place feature) might not be as perceptually salient as other segments. It is conceivable that its presence in the speech input might not be immediately taken into account in lexical access. Second, given that the surface phonetic form of a glottal stop has two sources in Maltese (prosodic-structurally driven or lexically driven), the competing lexical hypotheses could be activated to a comparable degree at the initial stage of processing. Subsequently, the higher variant frequency of the surface phonetic realization of an underlying glottal-stop (for the word with the underlying glottal-stop) may lead to lateral inhibition of the vowel-initial word for which the surface phonetic realization of the glottal stop has a lower variant frequency (50% compared to more than 95% for the underlying glottal stop-initial word).

In summary, Experiment 1 shows that the competition between vowel-initial words and glottal- stop-initial words is not modulated by the phonetic detail of the glottal stop in the speech input (whether it is hyperarticulated or not), which is in sharp contrast with the general assumption that lexical activation and competition are modulated by the phonetic granularity of speech input [see 34 for a review]. This finding can be interpreted in light of [15]’s view that a phonological variant with an initial glottal stop is stored for vowel-initial words in the mental lexicon. That is, the heightened phonetic clarity is likely to enhance the feature of such lexically represented glottal stop variants, activating the vowel-initial word to a similar extent that it activates the underlying glottal stop-initial word. A question arises here as to whether strong activation of the vowel-initial words by glottal stops is a consequence of the experimental design used in Experiment 1. As we mentioned in the introduction, epenthetic glottal stops with vowel-initial words occur about 50% of the time, and [15] further noted that speakers differ in their likelihood of using an epenthetic stop. However, in Experiment 1 of this study and in a similar experiment (Experiment 3) in [15], all the stimulus sentences contained a form of glottal stop for both the V-initial words and the /Ɂ/-initial words. So we cannot rule out the possibility that strong activation of the V-initial words is attributable to listeners’ learning of the distribution of epenthetic glottal stops across all the V-initial words used in the task. That is, because the V-initial words were consistently produced with an epenthetic glottal stop, listeners might have expected a glottal stop to be equally likely to be epenthetic or phonemic. In Experiment 2, we use a perceptual-learning paradigm to test this possibility by examining how strongly vowel-initial words compete for recognition with glottal- stop-initial words when participants hear a speaker who does not use epenthetic glottal stops.

## 3. Experiment 2

This experiment tests whether the strong activation of vowel-initial words in the presence of a glottal stop in the surface form, as observed in Experiment 1, can be attributable to the consistent occurrence of glottal-stop epenthesis for vowel-initial words. To this end, we use a perceptual-learning paradigm in which participants are first exposed to a particular speech pattern by a particular speaker, and the consequences thereof are investigated in a test phase [47]. Typically, participants receive exposure to a non-canonical phonetic pattern of a sound for at least 20 instances (e.g., tokens of /s/ produced in an unusual way) and are tested on other words containing that sound to examine the extent to which the unusual way of producing a particular sound has been learned. During exposure, participants can perform a lexical decision task [49] or simply listen to sentences or a story [48, 49]. The effects of exposure often are measured in a lexical-decision task [50, 51], or an eye-tracking task [19, 48].

In this experiment, we follow the design of [19], who investigated perceptual learning of variants in fluent speech in Dutch. In their case, the speaker either reduced a common Dutch prefix (/fəɹ/ → [f]) or produced a stop in an unstressed syllable as an approximant (/bi’kini/ → [ʋi’kini]. Participants looked faster at the intended word after being exposed to previous examples of that speaker performing those specific reductions. That is, one group was exposed to the prefix reduction (/fəɹ/ → /f/) and the other to the stop lenition (/b/ → [ʋ]), and then both groups were tested on both types of reduction. Each group outperformed the other when the target word carried the type of reduction to which the group had been exposed. This shows that the effects cannot be explained by an across-the-board adaptation to a casual speaking style that generally induces phonetic reductions. As in [19], participants of Experiment 2 were exposed first to 48 sentences, half of which contained a vowel-initial word with no epenthetic glottal stop and the other half of which contained a glottal stop-initial word with a full stop. After the exposure session, an eye-tracking experiment like the one in Experiment 1 was performed but with the modification that the vowel-initial words were never marked with an epenthetic glottal stop. That is, for glottal stop-initial words, the stimuli were the same as in Experiment 1, so that any differences must be a consequence of the exposure phase and/or the absence of an epenthetic glottal stop for the vowel-initial words during the test phase. As a necessary consequence, for vowel-initial words, the input signals in Experiment 1 and Experiment 2 differed, which means that differences are most likely attributable to the speech signal during the test phase rather than learning that might have occurred during the exposure phase.

We chose this design to answer three specific questions. First, by including some words in the test phase that were used in the exposure phase, we can investigate whether participants benefit from having heard a particular word produced by a given speaker in a specific way. Second, vowel-initial words should have less competition from glottal- stop-initial words in this experiment than in Experiment 1, simply because the test phase does not contain any glottal stop in the speech input signal. Third, glottal- stop-initial words should have less competition from vowel-initial words than in Experiment 1 when listeners learn during the exposure phase that vowel initial-words are unlikely to carry an epenthetic glottal stop.

### 3.1. Methods

#### 3.1.1. Participants

Forty students from the University of Malta participated in this study. They were aged between 18 and 32 years, and 27 of them were women.

#### 3.1.2. Apparatus and stimuli

The apparatus was the same as in Experiment 1, and the stimuli for the test phase used the same fillers and experimental items as in Experiment 1. The only difference was that glottalization was edited out for stimuli with vowel-initial targets. The non-glottalized parts of the vowels were increased in length by about 40 ms by repeating glottal cycles of the vowel, restoring the original vowel duration. Two glottal cycles from a labial nasal were spliced to the start of each vowel-initial word to allow a smooth transition from the precursors, which all ended on /m/ (i.e., /[t/j]ɪfhɛm/, Engl. ‘[she/he] understands’). For glottal stop-initial words, versions with a full glottal stop from Experiment 1 were used, and the amplitude of the /m/ was ramped down over the last 10 ms into the closure.

For the exposure, we generated 96 sentences with either a glottal-stop-initial or a vowel-initial word in a relatively predictable position (e.g., the word *awtur* in *Stephen King huwa magħruf bħala awtur ta’ kotba tal-biża*, Engl., ‘Stephen King is known as an author of horror books’; note that in this case, without the epenthetic glottal stop, the vowel hiatus is not resolved, a pattern that we had frequently observed [15]), because learning requires knowledge about the identity of a phonetic feature when hearing it [52]. These sentences were recorded multiple times by the same speaker who produced the experimental items, and one recording with no glottal stop for the vowel-initial words and a full stop for the glottal-stop-initial words was selected for use during the exposure phase. Half of the words used in the exposure phase were also part of the experimental list. Moreover, for half of them, we selected another word from the sentence for an attention test during exposure. This word was displayed in written form after the participant heard the sentence, and participants were asked to indicate whether the word was part of the sentence. For an equal balance of answers, half of these test words were replaced by semantic associates (e.g., *Awissu*, Engl., ‘August’ was replaced by *sajf*, Engl. ‘summer’).

#### 3.1.3. Procedure

Participants were first instructed—by text on the computer screen—to listen to the sentences during the exposure phase. They were told to listen attentively because, on some trials, they would be asked to indicate whether a given word was part of the sentence. After 48 trials of exposure, the test phase started.

The instruction for the test phase was the same as in Experiment 1 and was displayed on the computer screen after the exposure phase had finished. After reading the instruction, participants performed a nine-point calibration of the eye-tracker and then the 168 trials of the test phase (120 fillers and 48 experimental trials). The procedure was the same as in Experiment 1, with breaks after every 50 trials.

### 3.2. Results

#### 3.2.1. Exposure phase

The data from one participant was not usable because the data file generated by the eye-tracker was not readable (i.e., no ascii file could be generated). For the rest of the participants, the overall accuracy was 88% on the attention questions during the exposure phase (range: 56–100%), indicating that the participants paid attention during the exposure task.

For the results of the test phase, we examine the three questions stated above: are there repetition effects; do the competition effects for vowel-initial targets change depending on the input (with or without an epenthetic glottal stop); do the competition effects for glottal- stop-initial targets change depending on exposure (speaker consistently using or not using epenthetic glottal stops)?

#### 3.2.2. Repetition effects

**Fig 4:**
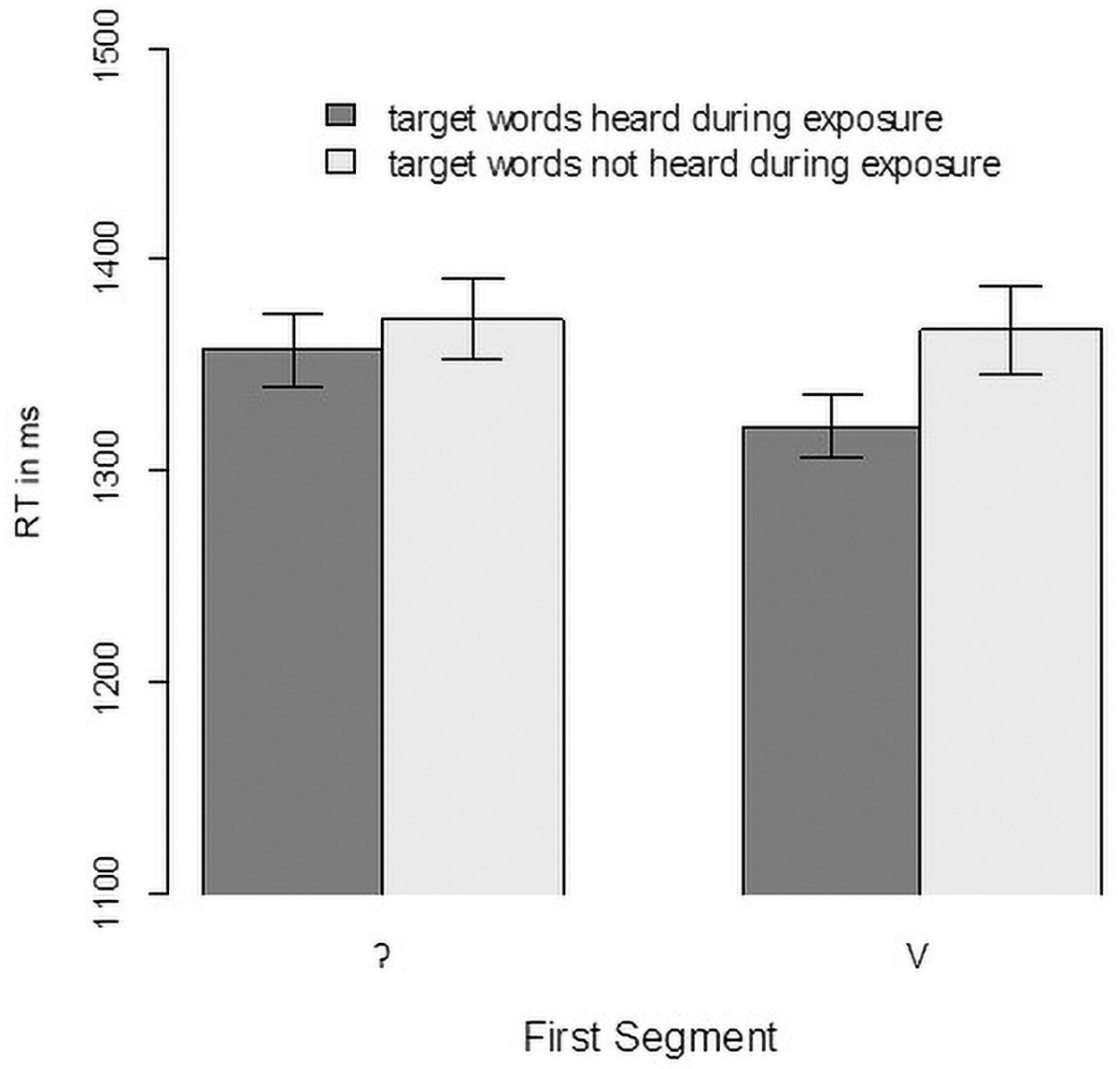
Mean reaction times in milliseconds in Experiment 2. Note that Mean reaction times (RT) and error bars were calculated on log(RT) and then transformed back to raw RT for easier readability. Error bars indicate the standard error for within-participant designs [53].

First, we analysed whether participants better recognized words if they had heard them during exposure. As in Experiment 1, accuracy was high, with condition means between 97.0% and 98.4%. With so few errors, we focused our analyses on the latencies of the click responses and the eye-movement data. As in Experiment 1, we first used an intercept-only linear mixed-effect model to filter reaction-time outliers (i.e., with a residual greater than 2.5 standard deviations from the mean considering participant and item random effects). That led to exclusion of 33 cases (1.8% of the total data). Fig 4 shows the mean reaction times for the remaining data, which are lower for items presented during exposure. This difference is slightly larger for vowel-initial words than for glottal stop-initial words.

The data were analysed with a linear mixed-effect model with reaction time (using its logarithm) as the dependent variable and contrast-coded predictors for the First Segment (-0.5 = vowel-initial, 0.5 = glottal-stop-initial) and Exposure (-0.5 = no, 0.5 = yes). Participants and items were used as random factors, and the maximal random-effect structure that led to convergence was used. In contrast to what Fig 4 might suggest, we found no significant effects (First Segment: b = 0.0135 SE—0.021, t(74) = 0.652, *p* = 0.517; Exposure: b = 0.019 SE = 0.012, t(1724) = -1.589, *p* = 0.112, interaction: b = 0.016 SE = 0.024 t(1730) = 0.661, p = 0.509).

**Fig 5:**
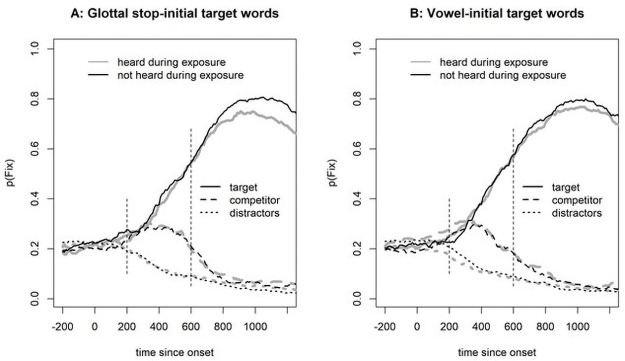
Fixation proportion in Experiment 2 relative to target word onset.

Turning to the eye-tracking data, Fig 5 shows the fixation proportions. There were no obvious differences between the conditions, and no significant effects were found in the linear mixed-effect model with target preference as the dependent variable, built according to the same principles as in Experiment 1 (First Segment: b = -0.094, SE = 0.251, t(94) = -0.37, p = 0.709, Exposure: b = 0.128, SE = 0.197, t(1805) = 0.65, p = 0.517, Interaction: b = 0.15, SE = 0.394, t(1805) = 0.38, p = 0.704).

#### 3.2.3. Vowel-initial words

**Fig 6:**
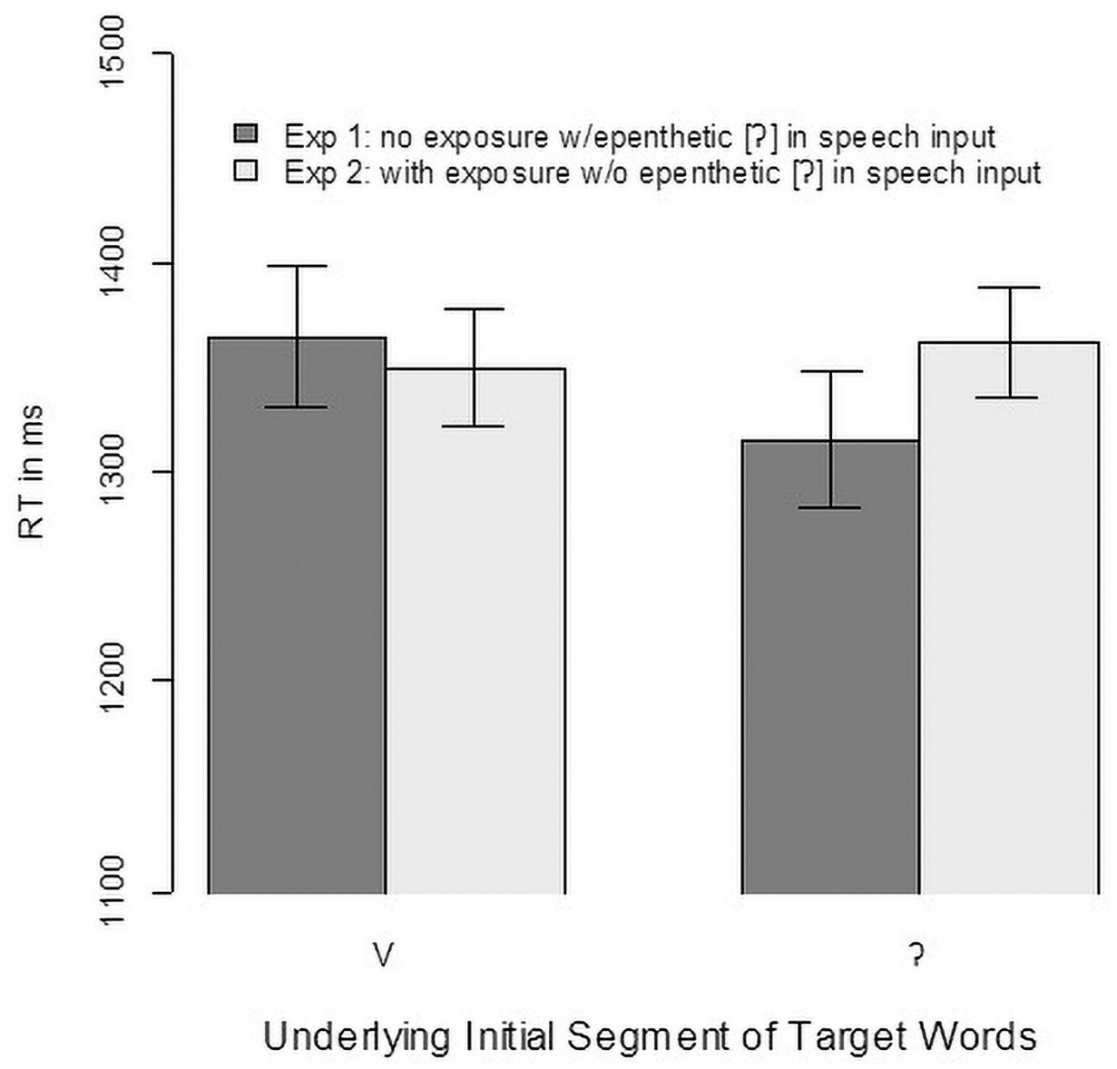
Reaction times from Experiment 2 compared with those from Experiment 1.

**Fig 7:**
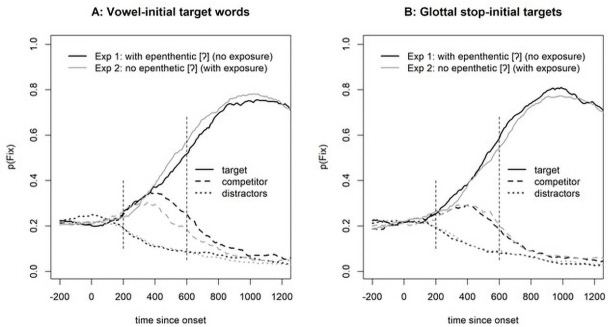
Eye-tracking results from Experiment 2 compared with those from Experiment 1. Note that in Experiment 1, vowel-initial targets were presented with an epenthetic glottal stop, and in Experiment 2, they were presented without such a stop. For glottal stop-initial targets, the signals in the two experiments were identical, but participants might have learned that a glottal stop was unlikely to be epenthetic, which would rule out vowel-initial targets from the onset-competitors.

Here, we compare the data from Experiment 1 to the data in the current experiment. For the vowel-initial words, the experiments differ in the input signals used. In Experiment 1, the vowel-initial words carried an epenthetic glottal gesture, whereas they were phonetically vowel initial (i.e., no glottalization) in Experiment 2. Fig 6 (left panel) shows the reaction time results, and Fig 7 (panel A) shows the eye-tracking results.

As Fig 6 (left panel) suggests, there is little difference in the reaction times, and the linear mixed-effect model with Experiment as the predictor (participants and items as random effects with the only possible slope of Experiment across items) reveals no significant effect of Experiment (i.e., whether the word was presented with or without an epenthetic glottal stop, b = -0.011, SE = 0.046, t(71) = -0.23, p = 0.817). The eye-tracking data (Fig 7, panel A), however, showed a difference, and the statistical analysis confirmed it (b = 0.403, SE = 0.204, t(1608) = 1.97, p = 0.049). Vowel-initial words suffered less competition from glottal stop-initial words when produced without an epenthetic glottal stop. It is noteworthy that the reduction in competition was surprisingly small. Moreover, the shape of the competitor function for Experiment 2 looked different than expected for competitors with a different initial segment [see, e.g., fig 7 in 9]. Usually, such competitors show fixation proportions that are initially—that is, about 200 ms after target onset—more like those of the distractors. But as Fig 7 shows, the competitors initially increase as sharply as do the targets. Usually, competitors with a different onset tend to show a slow increase only after 300 ms [see, e.g., fig 4 in 9]. In contrast, here we observed a sharp increase together with the targets up to 300 ms and then a decline, a shape typical for onset competitors.

#### 3.2.4. Glottal stop-initial words

We performed those same analyses for glottal- stop-initial words. As suggested by an inspection of Fig 6 (right panel), exposure to vowel-initial words produced without an epenthetic glottal stop (as in Experiment 2) did not lead to faster clicks on glottal stop-initial words (b = 0.037, SE = 0.044, t(69) = 0.82, p = 0.413). Similarly, as suggested by Fig 7 (panel B), the eye-movement data did not differ with and without exposure (b = -0.221, SE = 0.209, t(68) = -1.06, p = 0.295). This indicates that the listeners did not benefit from their exposure in terms of ruling out vowel-initial words as competitors for glottal-stop-initial words.

### 3.3. Discussion

Experiment 2 was set up to investigate three questions. First, we wanted to know whether there is a repetition effect such that words that have just been heard in a particular phonetic form are recognized faster when they are heard again a few minutes later. We did not find such a repetition effect. Given that repetition effects are considered among the most robust effects in the word-recognition literature [54], it seems unlikely that our failure to find such an effect would be due to lack of statistical power. With nearly 40 participants and 48 items, we are confident that our experiment was not underpowered [55]. Instead, our failure to find a repetition effect in Experiment 2 could lie in procedural differences between lexical decision tasks—which give rise to repetition effects—and those in the current study. In lexical decision tasks, participants are exposed to single-word utterances and required to react to every stimulus. That is, the repetition involves not only the stimulus, but also the response and the context (i.e., the word occurs as a single-word utterance on a given trial). In contrast, the repeated items in the current study were presented within different sentences during exposure and testing and required different reactions on the first and second encounters. During exposure, they were presented in a sentence that allowed some prediction to foster learning [46], and participants were listening and not responding. During the test phase, however, the repeated words required a reaction (a mouse click on the correct written word), and they were presented in non-constraining sentences that could contain a large number of words to visualize competition effects more clearly [see, e.g., 56, for how a biased context can decrease competition effects]. To summarize, robust repetition effects are observed in lexical decision tasks in which repetition includes the reaction and the context around the repetition of the critical word. Both of these aspects—context and reaction—varied across encounters in Experiment 2, which apparently seriously weakened the repetition effect. Nevertheless, given the descriptive (non-significant) trend for a repetition effect in the reaction-time data, it would be premature to rule out a potential repetition effect. However, given the current experimental settings, it is reasonable to argue that the repetition effect is rather small, if it exists, compared with what has been observed in lexical decision studies.

The second question was whether vowel-initial words would be recognized more easily, with less competition from glottal stop-initial words, when they were heard without an epenthetic glottal stop. We did find such a reduction in competition, with fewer looks to the glottal stop-initial competitor when the glottal stop was absent. Interestingly, however, the effect was small (just past the significance criterion), especially given that onset effects are usually very robust in eye-tracking experiments. We will return to this issue below.

Third, we asked whether activation of vowel-initial words when a glottal stop is heard would vary depending on whether the speaker makes use of epenthetic glottal stops. It did not: despite having heard all tokens of vowel-initial words with no epenthetic glottal stop, the results indicate that Maltese listeners still considered vowel-initial words as strong competitors for their glottal-stop-initial counterparts. This result is in line with [15]. If the phonological variant form of a vowel-initial word with a glottal stop is strongly represented in the mental lexicon, listeners might not learn to modify the lexical representation of a particular variant form as a consequence of consistent exposure to another variant type.

Our failure to find a learning effect can also be explained considering the general conceptualization of perceptual learning in speech [19, 30, 46, 51]. In perceptual learning studies, the effect of exposure on spoken-word recognition in the test phase is often interpreted as a retuning of pre-lexical representations. [19], for example, interpreted the learning effect as indicating the existence of pre-lexical representations of common prefixes, and [7] interpreted the finding of no generalization in learning from one allophone of the same phoneme to another as indicating the absence of phoneme-sized units. Given those considerations, a learning effect might require a pre-lexical unit that can “carry” the learning (see also [6]). The question then is whether an epenthetic glottal stop in Maltese is a pre-lexical unit. Mitterer et al. [15] showed that the primary acoustic correlates of the strength of the glottal gestures (presence of a full closure and duration) do not reliably distinguish epenthetic stops from lexical glottal stops. Based on that observation, they suggested that there is no bottom-up phonetic support for the existence of a pre-lexical unit for the epenthetic glottal stop. Thus, there is no “place” where such learning could occur in a way that generalizes it from one word to another.

Therefore, the most noteworthy finding from Experiment 2 might be the strong activation of glottal- stop-initial competitors by vowel-initial targets even when those targets do not carry an epenthetic glottal stop. As we noted in the results section, the shape of the competition function was unexpected, showing an unusual initial sharp increase in line with target–competitor pairs that share initial segments, as observed in [9]. One explanation for this pattern could be that the glottal stop has a special status in lexical processing in that it might not contribute strongly to the lexical activation of glottal stop-initial words, at least not as strongly as do other segments.

However, this is a comparison across experiments with slightly different paradigms. For instance, our current experiment contained no full rhyme competitors [such as beaker vs. speaker in 9]; our target–competitor pairs overlapped in only a few segments at the beginning of the word. Moreover, we are not aware of a study that directly investigates onset versus rhyme competitors in a language with a non-concatenative morphology, such as Maltese. Perhaps, onset differences in this type of language do not generally lead to strong *deactivation* of a competitor with an initial mismatch. Note that Onsets can often be morphemes, so that words that differ in onset (such as *jifhem* and *tifhem*; Engl., *he understands* and *she understands*, respectively) are the same word in a different conjugation. In the next experiment (Experiment 3), we explore this possibility by examining whether an onset mismatch caused by the presence or absence of another type of consonant will show competition effects similar to those observed with the glottal stop. For this test, we choose a voiceless alveolar stop /t/ because it is assumed to be unmarked in some phonological theories, so that it might not pose a severe recognition problem, possibly leading to some degree of activation of /t/-initial words in cases of mismatch [57; but see 58–60, for the limited utility of such theories for spoken-word recognition].

## 4. Experiment 3

In Experiment 3, we test whether onset mismatch generally leads to *deactivation* of competitors in Maltese. We hypothesize that the glottal stop is processed differently from oral stops, such that upon hearing a vowel-initial word, listeners would activate a /t/-initial competing word *much less* than a glottal-stop-initial word. We chose /t/ because it is often considered to be least specified in terms of its phonological features, which is then less likely to influence lexical activations than other consonants [e.g., 1]. The experiment was similar to Experiments 1 and 2 in investigating the competition between words that overlap initially (except for the presence or absence of an initial stop). That is, the previous experiments made use of pairs with initial pseudo-overlap such as *qabad* /ʔɑbɑd/ versus *abaq* /ɑbɑʔ/, in which words overlap in the sequence /ɑbɑ/ but are differentiated by the presence or absence of an initial glottal stop. In this experiment, we use similar pairs that differ in the presence or absence of an initial /t/ (e.g., *tabakk* /tɑbɑk:/ versus *abaq* /ɑbɑʔ/). Pairs were generated to have a similar amount of overlap; but where that was not possible, we used a slightly larger overlap for the /t/-initial words (see S1 Appendix). Note that pairs with greater overlap are likely to increase the activation of the competitor, working against our prediction that /t/-initial words will be activated to a lesser extent when listeners hear vowel-initial words than when they hear glottal-stop-initial words.

### 4.1. Method

#### 4.1.1. Participants

Thirty students from the University of Malta participated in Experiment 3. They were aged 18 to 34 years, and 19 of them were women. They were all native speakers of Maltese.

#### 4.1.2. Stimuli and procedure

The filler stimuli were the same as in Experiments 1 and 2. For the experimental trials, we generated new pairs with an initial pseudo-overlap (i.e., the overlap was counted after the initial /t/). The mean overlap between target and competitor was 2.7 segments (compared to 2.4 segments for the items in the previous experiments). The speech was recorded by the same native speaker of Maltese who recorded the stimuli for the previous experiments. The same sentence frames were used as before:

“[Matthew|Daniel|Mary|Jenny] [j|t]ifhem *TARGET*”

(Engl. ‘[Matthew|Daniel|Mary|Jenny] understands TARGET’).

From these recordings, four precursors, one for each name, were selected and cut to end on an upward-moving zero crossing of the nasal murmur. For the /t/-initial targets, a second version of each precursor was generated by adding a partly voiced closure with a duration of 81 ms after “[j/t]ifhem.” This was a typical duration of a closure for this speaker. The closure contained 8 cycles of voicing that progressively decreased in amplitude over 29 ms, followed by 52 ms of unvoiced closure. It is common for voicing from a preceding segment to leak into a (phonologically) unvoiced closure. Target words were cut out of their respective sentences at either the end of the nasal murmur of the /m/ for the vowel-initial words or at the release of the /t/ for the /t/-initial words and concatenated with the four different precursors. (Note that the precursors contained a closure for the /t/-initial stimuli).

With these stimuli, different random orders were generated. Each participant was confronted once with each target–competitor pair; across participants, it was varied whether the /t/-initial word was the target and the vowel-initial word the competitor or vice versa. Random orders were generated for all participants individually with the same constraints as in the earlier experiments (three initial fillers and counterbalancing of target and competitor positions across conditions).

### 4.2. Results

**Fig 8:**
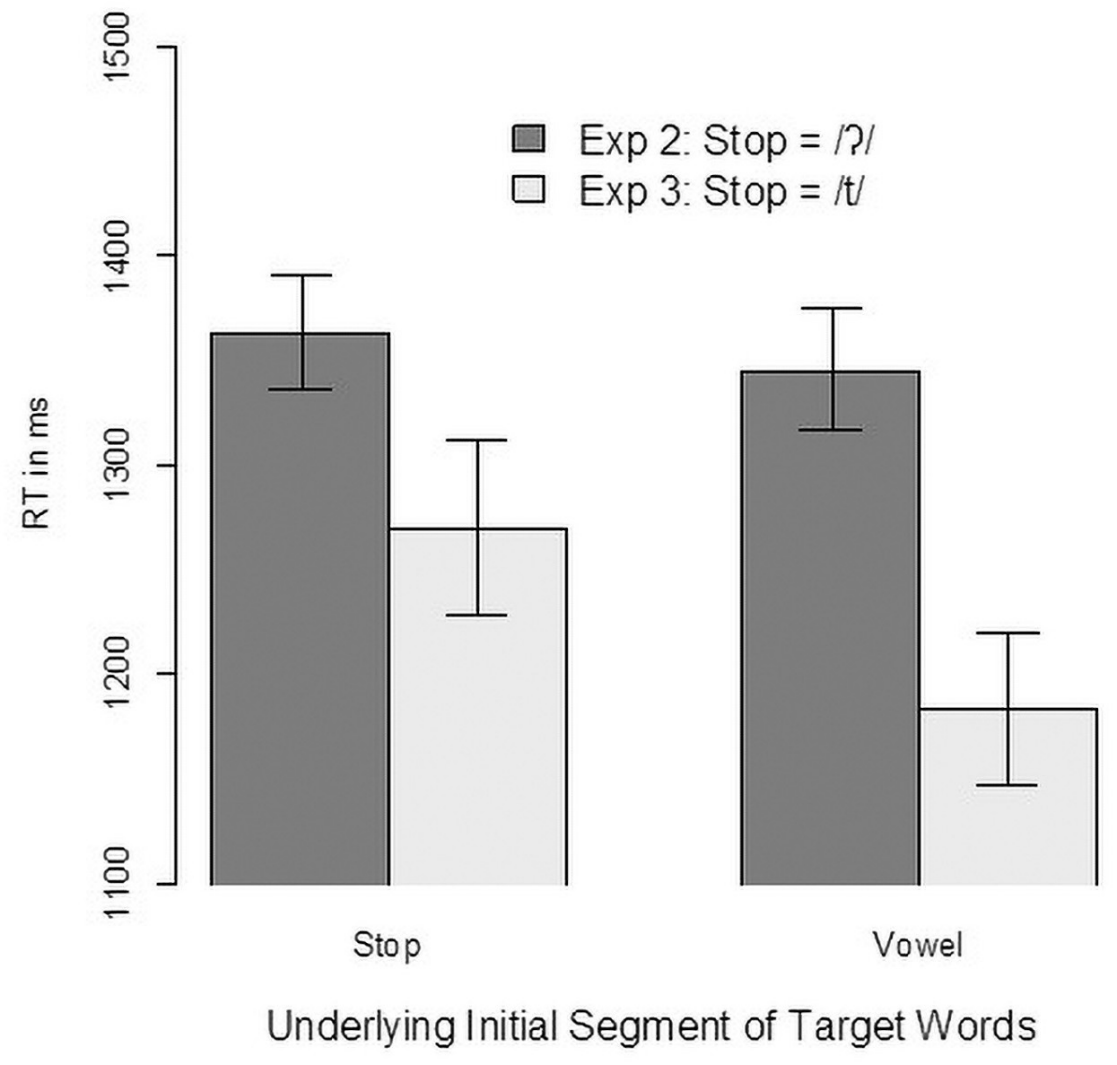
Reaction times from Experiment 3 (/t/-initial words) compared with Experiment 2 (/ʔ/-initial words).

As in the previous experiments, we found a high level of accuracy in the click responses (>99%). Analyses therefore focused on the reaction times of the click responses and the eye-movement data. Fig 8 shows the reaction times from Experiment 3 compared with the reaction times in Experiment 2. To analyse the reaction times, we first rejected unusual reaction times with an intercept-only model and then rejected trials with a residual larger than 2.5 standard deviations from the mean (53 trials, 1.7% of the data). For the remaining trials, we built a linear mixed-effect model with contrast-coded predictors for Initial Segment (vowel vs. stop, mapped onto -0.5 and 0.5, respectively) and Type of Stop (Exp 2: glottal stop, Exp 3: /t/, mapped onto -0.5 and 0.5 respectively). Random factors were participants and items. Even though there was some overlap in the vowel-initial items, we considered each item in its experiment as a unique level of the random-effect variable, with the item paired with a different competitor. This also means that there is only one possible random slope (initial segment over participants), which had to be trimmed to achieve convergence. The final model revealed a significant effect of Initial Segment b = 0.039, SE = 0.013, t(196) = 3, p = 0.003), a marginal effect for Type of Stop (b = -0.102, SE = 0.053, t(69) = -1.93, p = 0.058), and a significant interaction of the two factors (b = 0.053, SE = 0.026, t(196) = 2, p = 0.047). To further investigate this interaction, we built separate models to estimate the effect of Type of Contrast for both initial segments. For stops, we found no significant difference in the reaction time depending on Type of Contrast (b = -0.074, SE = 0.055, t(72) = -1.35, p = 0.182), but we did find an effect for vowels (b = -0.13, SE = 0.054, t(72) = -2.4, p = 0.019). That is, participants clicked faster on the vowel target when it was accompanied by a /t/-initial competitor than when it was accompanied by a glottal- stop-initial competitor, which can be interpreted as evidence that /t/-initial words are less strongly activated than glottal- stop-initial words when the initial segment is missing.

**Fig 9:**
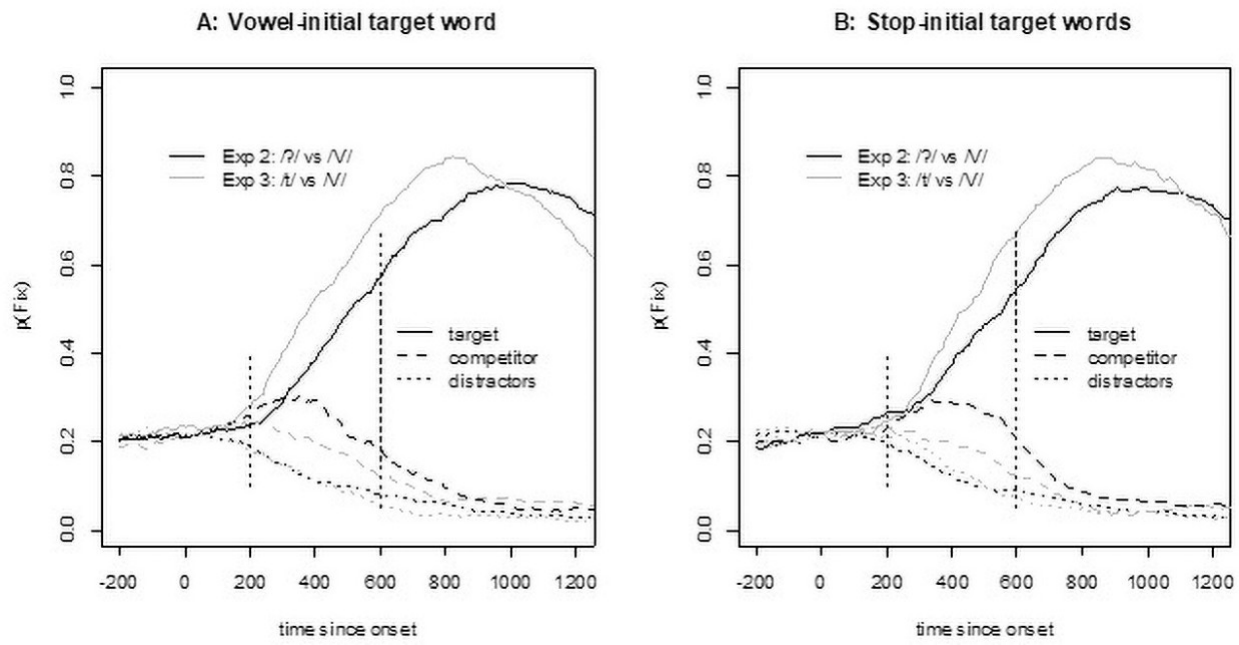
Eye-movement data from Experiment 3 (/t/-initial words) compared with Experiment 2 (/ʔ/-initial words).

Turning to the eye-tracking data, Fig 9 shows clear differences in the competition patterns for pairs in which the stop-initial word starts with /t/ compared with those in which it starts with a glottal stop. As discussed above, the competition pattern with glottal- stop-initial words contrasted with vowel-initial words has the shape typically seen with onset competitors. In contrast, the competition between /t/-initial words contrasted with vowel-initial words looks more like the pattern expected for rhyme competitors. This finding was confirmed in a linear mixed-effect analysis for the target preference in the time window of 200–600 ms after target onset (the same window as in all other analyses). The model used contrast-coded predictors for Initial Segment (vowel vs. glottal stop, mapped onto -0.5 and 0.5, respectively) and Type of Contrast (Exp 2: /ʔ/ vs /V/, Exp 3: /t/ vs /V/, mapped onto -0.5 and 0.5, respectively) as fixed effects and participants and items as random effects. The model with the random slope of Initial Segment over participants (the only possible random slope) did not converge, and the models with random intercepts only revealed a significant effect for Type of Stop (b = 1.033, SE = 0.212, t(79) = 4.88, p < 0.001). Initial Segment had no significant effect (b = -0.218, SE = 0.171, t(206) = -1.27, p = 0.205), nor did the interaction (b = -0.245, SE = 0.342, t(206) = -0.71, p = 0.476).

**Fig 10:**
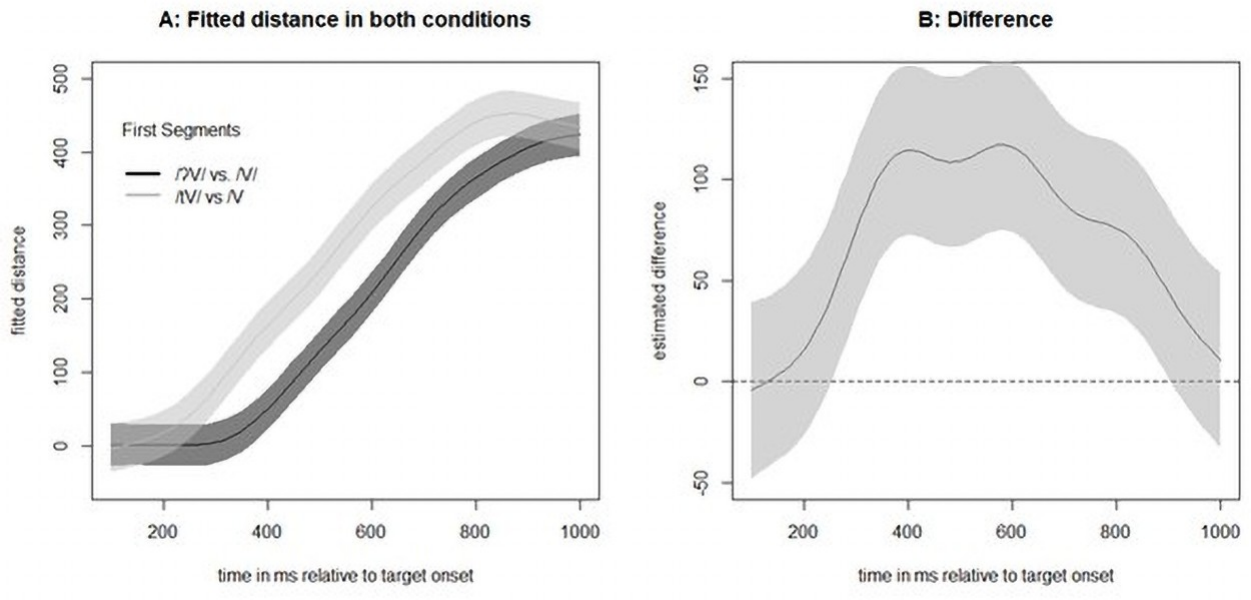
Outcome of the general additive model for target preference comparing vowel-initial words with stop-initial words (/t/-initial or /ʔ/-initial).

To further substantiate this difference in competition pattern (/tV/ vs /V/ initial words), we compared it with competition pattern from Experiment 2 (/ʔV/ vs /V/ initial words) using a GAM similar to the one in Experiment 1 with a capped target-competitor distance measure as the dependent variable. We first calculated a model with Experiment as a fixed factor, including a smooth term, and two random effects for participants and items with smooth terms. Item was coded as a target–competitor pair, so a different random effect was calculated when the target word was the same but the competitor was different. Therefore, additional random slopes were not possible because the fixed factor Experiment is a between-participant and between-item variable. The resulting fit (see Fig 10) showed that the two conditions differ relatively early, 250 ms after target onset, which is the time range expected for differences between onset and rhyme competitors.

### 4.3. Discussion

The results of Experiment 3 show that the unusual onset mismatch effect observed with a glottal stop is not found with the oral stop /t/, indicating that the onset mismatch involving an oral stop in Maltese does lead to the generally expected *deactivation* of competitors. More specifically, even though glottal stop-initial words are strongly activated even when the glottal stop is not present in the speech input (Experiment 2), the results of Experiment 3 show that this is not the case for oral stops, even when the studies are designed in a similar fashion. There is a clear difference in the competition patterns between vowel-initial words on the one hand and stop-initial words on the other hand, depending on whether that stop is oral or glottal. The results, therefore, are consistent with the hypothesis that glottal stops are processed differently from oral stops in Maltese, in that they do not constrain lexical activation as strongly as do other oral segments.

## 5. General discussion

In a series of experiments, we have explored issues related to how words with an initial glottal stop can be processed in Maltese, a language in which the glottal stop is used both as a phoneme and as an epenthetic stop. In Experiment 1, we explored whether and how the fine phonetic detail of a glottal stop that arises with hyperarticulation (with a full glottal adduction gesture) would influence lexical completion between two lexical hypotheses whose segmental make-ups overlap substantially, as in *qattus* (glottal stop-initial) and *attur* (V-initial). The heightened phonetic clarity of a hyperarticulated form is generally assumed to enhance the phonologically distinctive features of a segment, which maximizes its lexical distinction [24–27, 34]. Based on this assumption, we hypothesized that a hyperarticulated phonetic form of the glottal stop would lead to stronger activation of words with an underlying glottal stop (specified with a glottal adduction feature) as compared with vowel-initial words for which a glottal stop is epenthesised to serve as a phonetic marker of a prosodic boundary. However, our results show no evidence for this, implying that the hyperarticulated form of the glottal stop does not necessarily enhance the underlying distinctive feature but instead leads to activation of the competing lexical hypothesis (i.e., a vowel-initial word) to a comparable degree. It appears that listeners’ performance reflects the production statistics that epenthetic glottal stops, if they occur, are not on average phonetically any weaker than underlying glottal stops.

In Experiment 2, we explored whether activation of both glottal stop-initial and vowel-initial words in the presence of a glottal stop could be due to the distribution of the glottal stop in the stimuli—i.e., all the vowel-initial target words in Experiment 1 (and in [15]) were presented consistently with an epenthetic glottal stop. We tested this possibility by examining whether listeners can adapt to a particular speaker’s production pattern if the speaker does not use a glottal stop epenthetically for vowel-initial words, so that all glottal stops encountered indicated an underlying glottal-stop-initial word. The results did not reveal any evidence for that possibility, showing that a listener’s exposure to a particular speaker’s use of a glottal stop does not modulate the competition. That is, not only are vowel-initial words still activated strongly with a glottal stop in the input signal, but also underlying glottal- stop-initial words are activated even in the absence of a glottal stop in the input signal. This again implies that the onset competition between words that involve a glottal stop operates differently from the general onset competition effects that are modulated early in the spoken word recognition process by acoustic mismatches (e.g., [9, 10]). The failure of the general onset competition effect, however, is not a rarity if we consider that lexical competition is often modulated by factors other than the amount of segmental overlap between lexical hypotheses. For example, the reliance on segmental overlap in determining the (de)activation of a competitor can be reduced by extraneous noises [14] or in casual speech [13]. Thus, the observed reduction of the listeners’ reliance on the onset overlap in Maltese may not be entirely unusual. In fact, we cannot rule out a possibility that such reduced reliance on the onset overlap for lexical assess may be a processing characteristic of a Semitic language (with a non-concatenative morphology).

In Experiment 3, we attempted to test this possibility by addressing the specific question of whether the reduced processing role of the onset mismatch observed with a glottal stop was generalizable to a case with an oral stop. To do that, we contrasted vowel-initial words with words starting with an oral stop /t/ which is often considered to be unmarked, possibly imposing fewer constraints on speech perception than other segments [see, e.g., 57, see 61, for a related discussion]. Our results show that, unlike the case with a glottal stop, Maltese listeners heavily weigh the onset mismatch (the presence or absence of an oral stop) in lexical access.

The converging evidence from the results of our three experiments suggests the special status of a glottal stop in lexical processing—i.e., a competition between words with an initial glottal stop is not as strongly regulated by the onset acoustic mismatch as a comparable lexical competition of words with an oral stop. In other words, glottal stops in the word-onset position do not constrain lexical access as strongly as do other segments, if at all. Our results have implications for two questions. First, how are variant forms stored in the mental lexicon? Second, what is the status of the glottal stop in the phonological system of Maltese and other languages that use it as a phoneme?

### 5.1. The multiple representation account: Multiple phonetic variants are stored in the mental lexicon

Our results have theoretical implications for how words are stored in the mental lexicon and what kinds of information can be stored for the words. As we discussed earlier in the paper [15], suggested that strong activation of both glottal stop-initial and vowel-initial words (failing to show a usual onset acoustic mismatch effect) can be interpreted to indicate that the phonological variant form in which a vowel-initial word has a glottal stop is stored in the lexicon. This is also in line with the view that listeners store information about patterns of variation [62] or multiple forms of the same word [see, e.g., 5] in the lexicon. This study builds on the literature by presenting a case in which a seemingly non-canonical variant that deviates from an underlying form (in this case, a vowel-initial word) also can be strongly represented in the lexicon. For example, as we discussed above, a hyperarticulated form provides comparable phonetic support for both underlying glottal-stop-initial words and vowel-initial words, indicating that phonetic enhancement of a glottal adduction is processed in reference to both lexical hypotheses. This possibility could be buttressed by the listeners’ failure to learn the distributional patterns of a given speaker. It is conceivable that listeners are less likely to adjust their perception to a particular speaker’s production pattern (of a vowel-initial form) when multiple representations (i.e., vowel-initial and glottal stop-initial forms) are stored in the lexicon, presumably with equal strength. (See below for further elaboration on this point).

The present study, however, was not designed to directly evaluate different theoretical positions. For example, our results do not provide direct evidence for the detailed phonetic granularity of what is stored in the mental lexicon for glottal stop-initial words versus vowel-initial words—i.e., whether it takes the form of abstraction [see 1–6, 8] or a gradient of fine phonetic detail for all possible exemplars [see, e.g., 2–4]. Nevertheless, our results shed some light on that debate. For example, that the fine phonetic detail of hyperarticulation does not induce activation differences suggests that information about glottal stops, whether underlying or epenthetic, is coarsely represented in the lexicon rather than being fine-grained. On the basis of that interpretation, our results are in line with the view that listeners store multiple abstract forms of each word [e.g., 5], which could also be in the form of allophones [7, 8].

### 5.2. The prosody account: A glottal gesture is prosodic in nature

One way to explain the weak modulation of competition by the form or even the presence or absence of a glottal stop is that the glottal gesture, whether underlying or epenthetic, could be prosodic in nature and does not constrain lexical access as do other supralaryngeal segmental features. Considering a glottal gesture to be prosodic might not be immediately transparent because glottalization is not generally included in the set of prosodic features such as pitch, duration, and amplitude. However, as discussed in [63], there is ample evidence that glottalization (often called laryngealization) can co-occur with other prosodic events such as stressed syllables, tonal accents, or sentence intonation (particularly with low tones) across many languages. This is precisely in line with the proposal made by Garellek [64] that glottalization of a vowel-initial word (as evident in English and Spanish) serves to enhance prominence of stressed vowel-initial syllable of a word. Thus, Garellek’s proposal implies that V-initial glottalization is prosodic in nature in that it helps marking prominence of the prosodic structure. Here we extend this prosodic function of glottalization to phonetic realization of an underlying glottal stop that has been traditionally considered as segmental.

Moreover, glottalization, a frequent phonetic form of the glottal stop, is often realized as a drop in F0 and amplitude, two features typically associated with prosody. There is also a phonological approach to representation of glottal gestures that resonates with this possibility. Kehrein and Golston [65] observed that the occurrence of laryngeal features (e.g., for voicing, aspiration, and glottalization) is restricted, so that no more than two laryngeal features can occur per syllable constituent (onset, nucleus, coda), and these features are unordered. Based on these observations, they propose that laryngeal features, including a glottalization feature, are not properties of segments but of the prosodic structure of the syllable. In this view, laryngeal features are licensed by prosodic structure, and never analysed as segments, largely in line with the proposal made by [64]. In a similar vein, Garellek et al [36] suggested that while treating a glottal stop as a segment may be useful for phonological analysis, the actual phonetic realization of a glottal stop (or glottalization) can be better characterized as modulation of phonation gesture (with a range of phonetic implementation that may vary with prosodic structural factors), which poses challenges for treating it as segmental.

Although our data support such a claim, the root morphology in Maltese provides a challenge to this theoretical approach [65]. The root morphology in Maltese allows the first two root consonants in the onset position to indicate some past tense forms (e.g., *k-t-b*, the root for ‘writing,’ gives rise to *ktibt*, Engl., ‘I wrote’). Interestingly, however, the order of the two consonants is often contrastive when the onset contains one oral and one glottal stop (e.g., *qbadt*, Engl., ‘I caught’ versus *bqajt*, Engl., ‘I stayed’). At first glance, this could appear to run counter to [65]’s proposal. It remains to be seen whether the different orderings (e.g., /bʔ/ versus /ʔb/) lead to different perceptual consequences or if the contrast is recovered only through the context. Nonetheless, a glottal stop in such a case might still be considered prosodic. Kiparsky [66] provides a formal account that some Arabic dialects (which in his definition includes Maltese) allow those consonants to form their own semi-syllables that stand outside the syllable, so that a glottal stop is analysed as extra-syllabic or prosodic, which is in principle consistent with the prosody account of [65]. One weakness of [66]’s approach, however, is that it fails to predict how extra-syllabic consonants differ from syllabic ones. This would be an avenue for further research.

Based on those phonetic and phonological considerations, it is reasonable to propose that the prosodic characteristics of a glottal stop in production (with a laryngeal specification of glottal adduction) are mirrored in perception, so that the glottal stop is analysed in reference to prosodic structure. In fact, the prosodic account being proposed here runs somewhat counter to our recent discussion about the role of the Maltese glottal stop in speech processing [67]. In [67], we provided evidence that Maltese listeners can use an epenthetic glottal stop as a cue to prosodic structure in a language in which it also serves a segmental function as an underlying phoneme (unlike in English and other languages in which a glottal stop is always prosodic-structurally driven). So, in [67], the data were interpreted based on an *a priori* assumption that information about a glottal stop must be processed as being primarily segmental, given that the glottal stop is part of the segmental inventory of that language. The prosodic account being proposed here departs from that assumption and explores an alternative possibility under the assumption that a glottal stop is intrinsically prosodic. For a sound to be prosodic does not mean that its information is not stored in the mental lexicon. On the one hand, information about the glottal stop as a prosodic feature could be stored along with other segmental information about the words, just as information about tonal (prosodic) features is stored in the mental lexicon in a tone language. Mitterer and Reinisch [68] showed that words with a lexical glottal stop in Maltese are recognized less efficiently when the glottal stop is absent. This indicates that the glottal stop is part of the lexical representation of words. On the other hand, for vowel-initial words, information about the glottal stop can be stored in the lexicon as phonetic variants. Listeners can then retrieve stored information about the glottal stop to distinguish words by referencing prosodic structure. If the computed prosodic structure licenses the glottal information in a particular prosodic position (e.g., in an initial position of prosodic words), a comparable degree of activation could arise across lexical hypotheses that contain the matched information, regardless of whether the information comes from an underlying or epenthetic glottalization feature.

This prosodic account is compatible with the multiple representation account that we discussed above. Both assume that phonetic variants with a glottal stop are stored in the lexicon, so that simultaneous activation of vowel-initial and glottal- stop-initial words is no longer seen as deviating from the usual acoustic mismatch effects. But the prosodic account further assumes that the lexically stored prosodic information (i.e., the glottalization feature) is processed by a prosody analyser in reference to prosodic structure, which is computed in parallel with segmental information [see 20 for the *Prosody Analyser* account]. One way to determine whether an effect comes about as a consequence of a segmental analysis versus a prosodic one is by analysing the time course of lexical access, as suggested in recent studies [22, 23, 59, 69]. These studies indicate that listeners generally integrate the use of prosodic information later in the spoken-word recognition process.

Here we recapitulate two specific findings of this study that lend support to the prosodic account. The first is that the hyperarticulated form of the glottal stop does not lead to any activation difference between lexical hypotheses. This can be interpreted in terms of [22]’s “two-stage” model of the *Multistage Assessment of Prominence in Processing*. The model assumes that segmental strengthening (i.e., hyperarticulation) that can be caused by prominence facilitates segmental analysis, and that the effect occurs early in the time course of lexical access, whereas prosodic cues to prominence come into effect at a later stage of lexical processing. Evidently, the absence of such an early effect with a glottal stop indicates that its acoustic cues do not enter early into segmental processing. The second related finding comes from our detailed analysis of the time course of lexical access (see the GAM analysis for Experiment 1). It reveals evidence that, at a later stage in processing, listeners do interpret a glottal stop as being associated with an underlying glottal- stop-initial word. This supports the view that the glottal stop is prosodic in nature and is consistent with the general assumption that prosodic analysis comes into effect at a later stage of lexical processing [15, 22, 23, 67, 69].

Assuming that the glottal stop is naturally prosodic provides an alternative perspective on how to explain the initial parallel activation of vowel-initial and glottal stop-initial words, with a subsequent preference for the glottal stop-initial word shown in the GAM analysis for Experiment 1. If multiple phonetic variants of vowel-initial words (including a glottal-stop-initial form) are stored in the lexicon, there is no *a priori* reason to predict that the acoustic cues to a glottal stop are eventually used to more strongly support words with an underlying glottal stop. The assumption of lexical storage of a glottal stop variant was needed to explain the strong activation of vowel-initial words with an initial glottal stop. However, if that glottal stop is not strongly affecting lexical access, this assumption might not be necessary. In other words, we could modify our initial assumption to posit that the stored information is more parsimonious, including only information for the underlying glottal stop, whereas the epenthetic stop is assigned post-lexically. In that case, the acoustic cues for the initial segments are likely to activate both words that contain the matched acoustic information stored in the lexicon, but when the prosodic analysis is brought to bear on the lexical hypothesis, vowel-initial words assigned a glottal stop post-lexically are at a disadvantage.

Before we present the conclusion, it should be noted that our proposals are not conclusive. Many issues remain to be resolved before we can reach a firm conclusion. First, although this study shows evidence for a later use of glottal stop information, similar eye-tracking experiments in [15] did not show the same effect. Future work is called for to provide more solid ground for the prosody account. Second, in Malta, English is an official language, so Maltese speakers use Maltese English as a second language. Furthermore, given its geographical location, Maltese speakers are frequently exposed to Italian. These language contexts leave open the question as to whether the observed effect is a unique case of Maltese, or if it can be generalized to other Semitic languages that use glottal stops in a similar way. Again, to address this issue, further research is needed to compare our results with data to be obtained from other Semitic languages. Third, it remains to be seen how taking the glottal feature as prosodic compares with the effects of other, more traditionally defined prosodic features such as tone and duration. The data from [70], for example, show no strong time course difference in processing tone and segmental information in Mandarin. Although they showed that tone effects can come into play somewhat later in processing and can easily be explained by the tone contour needing more time to be fully distinguishable (just as vowel perception is influenced by durational information later than by spectral information, see [39]). Thus, different prosodic features can be processed differently in lexical processing. Again, more work is needed to determine how specific prosodic features are processed at different stages in connection with lexical access.

In conclusion, the data presented in this study provide converging evidence that a glottal stop in Maltese is processed differently from other (oral) segments. In particular, the presence or absence of a glottal stop does not appear to constrain lexical access in the way that other segments do. We consider two possibilities: the multiple representation account and the prosodic account. Both accounts assume that multiple phonetic variants, including epenthetic glottal stops for vowel-initial words, are stored in the mental lexicon, such that the apparent onset mismatch between an underlying glottal- stop-initial word and a vowel-initial word does not contribute to lexical competition at an early stage of speech processing. For the prosodic account, we further assume that the glottal stop or glottalization feature is prosodic in that its effect enters into speech processing at a later stage which involves prosodic analysis. Although further research is certainly called for to corroborate the proposals that have been made here, this study showed that both segmental and prosodic information might be stored in the mental lexicon, depending on the phonetic content of the sound. More broadly, there is a growing body of literature that views prosodic analysis as an integral part of speech comprehension and provides some basis from which theories of speech perception can develop to devise mechanisms for integrating segmental analysis and prosodic analysis, which might operate in parallel but at multiple stages.

## Supporting information

S1 Appendix A list of stimuli used in the experiments. (DOCX)
