# Supplementary material for: Glottal stops do not constrain lexical access as do oral stops
Source: PLoS One. 2021 Nov 19;16(11):e0259573. doi: 10.1371/journal.pone.0259573 (PMC8604299; doi:10.1371/journal.pone.0259573)
Supplement: S1 Appendix — (DOCX) [file pone.0259573.s001.docx]

Appendix. A list of stimuli used in the experiments.

| Experiments 1 and 2 | |  | Experiment 3 | |
| --- | --- | --- | --- | --- |
| Glottal stop-initial word | Vowel-initial word |  | /t/-initial word | Vowel-initial word |
| qabad | abaq |  | tabakk | abaku |
| qabar | għabra |  | tabal | għabra |
| qabbad | abbati |  | tabbabi | abbati |
| qabel | abjad |  | tabib | abjad |
| qabeż | għabbex |  | tabba' | għabbex |
| qabru | abbuż |  | tabbal | għabba |
| qaddej | addoċċ |  | tabella | appella |
| qaddiefa | għaddas |  | tajjeb | għajjeb |
| qaddisa | addotta |  | taxxa | axxa |
| qadef | adda |  | tadam | għadam |
| qadima | għadira |  | tavla | għażla |
| qafas | affari |  | taffej | affett |
| qafla | afda |  | tagħna | għana |
| qaħba | aħbar |  | taġen | għaġen |
| qajjem | ajruport |  | tazza | għadsa |
| qala' | għalqa |  | talba | għala |
| qalb | Alpi |  | talb | għall |
| qalziet | alkoħol |  | taqta | aqta |
| qamar | amment |  | tamma' | għammar |
| qamħ | alf |  | tard | għarb |
| qammiela | ammetta |  | tinxef | infexx |
| qanfud | anzjan |  | timbru | imperu |
| qanpiena | annimal |  | tetnu | għentu |
| qarabagħli | aranċina |  | temtem | emmen |
| qarben | għarbiel |  | tarbija | għarbija |
| qarn | art |  | tinda | inka |
| qarnit | armata |  | tombla | ombra |
| qarrej | ardit |  | tiben | iben |
| qasab | assalt |  | tassew | għassew |
| qasrija | għasfur |  | tasfa' | asfalt |
| qassata | assistent |  | torċa | orgni |
| qastan | astrat |  | torri | orfni |
| qatel | attent |  | tattika | attakka |
| qatra | atleta |  | tagħrief | għaraf |
| qattus | attur |  | tempju | embriju |
| qawl | awtur |  | tawes | għaddes |
| qawsalla | Awstralja |  | tamal | għamel |
| qawwi | Awissu |  | tordna | ordni |
| qawwies | għawwiem |  | tambur | ambra |
| qiegħdin | editur |  | terra | erbgħa |
| qishom | isqof |  | tarmak | armajt |
| qodma | għodda |  | turban | urban |
| qoffa | offra |  | telf | elf |
| qorti | ordni |  | torti | għorka |
| qoxra | għoxrin |  | tonn | għonq |
| quċċija | uċuħ |  | torba | għorfa |
| quddiem | udjenza |  | tgħadda | għadu |
| qurdiena | urġenti |  | turrun | uranju |
